# Integrin alphavbeta3 enhances β-catenin signaling in acute myeloid leukemia harboring Fms-like tyrosine kinase-3 internal tandem duplication mutations: implications for microenvironment influence on sorafenib sensitivity

**DOI:** 10.18632/oncotarget.9617

**Published:** 2016-05-26

**Authors:** Hai Yi, Dongfeng Zeng, Zhaohua Shen, Jun Liao, Xiaoguo Wang, Yao Liu, Xi Zhang, Peiyan Kong

**Affiliations:** ^1^ Department of Hematology, Xinqiao Hospital, Third Military Medical University, Chongqing, 400037, People's Republic of China; ^2^ Department of Hematology, General Hospital of Chengdu Military Region, Chengdu, 610083, People's Republic of China

**Keywords:** integrin, acute myeloid leukemia, Fms-like tyrosine kinase-3 internal tandem duplication, β-catenin, drug sensitivity

## Abstract

Binding of leukemia cells to the bone marrow extracellular matrix (ECM) through integrins might influence drug response and the survival of acute myeloid leukemia (AML). However, the functions of integrin in AML are needed to be clarified. Data from The Cancer Genome Atlas (TCGA) were retrieved and integrin β3 (ITGB3) expression and prognostic significance for AML were analyzed. Integrin alphavbeta3 (αvβ3) in sorafenib sensitivity and signaling pathway of *FLT3-ITD* AML cells was evaluated *in vitro*. The level of ITGB3 expression was positively correlated with risk stratification and prognosis of AML patients, especially in cytogenetic-normal patients with Fms-like tyrosine kinase-3 internal tandem duplication (*FLT3-ITD*) mutation. Integrin αvβ3 decreased sorafenib sensitivity when co-culture of MV4-11 cells and bone marrow stromal cells (BMSCs), and it is crucial for osteopontin (OPN) induced sorafenib insensitivity in *FLT3-ITD* mutated AML cells. Mechanically, αvβ3 enhance β-catenin activation through phosphatidylinositol 3-kinase (PI3K)/Akt/Glycogen synthase kinase-3 beta (GSK3β) pathway. Moreover, genetic inhibition of β-catenin by shRNA could increase sorafenib sensitivity in MV4-11 cells. Taken together, our study revealed a novel mechanism in microenvironment influence on sorafenib sensitivity in AML with *FLT3-ITD* mutation that was caused by activating integrin αvβ3/PI3K/Akt/GSK3β/β-catenin pathway. Integrin αvβ3/β-catenin could be considered as a new therapeutic target for AML especially for *FLT3-ITD* mutated AML.

## INTRODUCTION

Great progress has been achieved in AML treatment through past decades, however, some types of AML, especially AML with Fms-like tyrosine kinase-3 internal tandem duplication (*FLT3-ITD*) mutation, which comprised of 20-30% AML [1], are still incurable using currently approaches except for allogeneic hematopoietic stem cell transplantation [2]. Tyrosine kinase inhibitors (TKIs) emerging as targeted therapy appeared most exciting therapeutics in *FLT3-ITD* treatment, such as multikinase inhibitor sorafenib [3] and some new compounds [4]. Although some TKIs could achieve high remission rate both in peripheral blood and bone marrow, leukemia cells usually relapse in a short period of time [5]. One reason is that TKIs execute subclone selection *in vivo*, amplifying drug insensitive clones, such as FLT3 point mutation (D835Y) clone [6]. Another reason is that drug resistant leukemia stem cells (LSCs) were protected from bone marrow niche, leukemia then re-established from LSCs under certain conditions such as TKI withdraw [7].

Leukemia/microenvironment interaction is important for leukemia cells especially leukemia stem cells' biological behavior [8]. Accumulating evidences indicate that integrins are involved in leukemia/microenvironment interaction by influencing both cell growth and drug sensitivity [9]. For instance, interaction of α4β1 and α5β1 in leukemia cells with fibronection in stromal contributes to chemo-insensitivity and minimal residual disease of AML [10, 11]. Integrin β could directly interact with integrin-linked kinase (ILK), which could trigger downstream signaling pathway, such as PI3K/Akt pathway, resulting in cell survival and proliferation [12]. Recently, an *in vivo* small hairpin RNA (shRNA) screening approach was used to find out integrin β3 was required for leukemogenesis and LSCs transcriptional programs maintenance in a MLL-AF9 AML mouse model. They also found that integrin αv, which formed a heterodimer with integrin β3, also required for maintaining the leukemic phenotype [13].

In this paper, we demonstrated that the expression level of integrin β3 was associated with the National Comprehensive Cancer Network (NCCN) risk stratification and could be a novel prognostic biomarker in AML especially in cytogenetic-normal *FLT3-ITD* mutated AML patients. Integrin αvβ3 decreased sorafenib sensitivity when co-culture MV4-11 cells with BMSCs, and it is crucial for OPN induced sorafenib insensitivity in *FLT3-ITD* mutated AML cells. αvβ3 could enhance β-catenin activation through PI3K/Akt/GSK3β pathway. Our study revealed a novel mechanism in microenvironment influence on sorafenib sensitivity in AML with *FLT3-ITD* mutation.

## RESULTS

### Relationship between ITGB3 expression and clinicopathological features of AML patients

**Figure 1:**
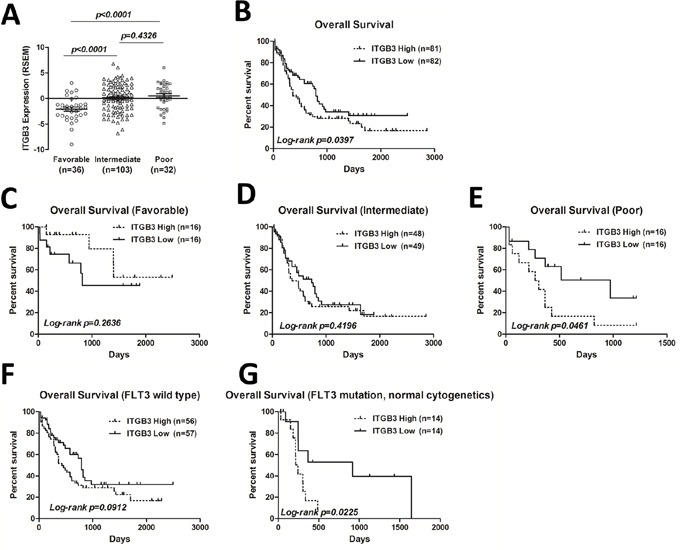
ITGB3 expression and correlation with overall survival (OS) in AML patients **A.** ITGB3 expression level in 171 AML patients from TCGA dataset (https://tcga-data.nci.nih.gov/tcga/) was grouped by cytogenetic risk according to NCCN. The expression level is represented by RSEM (RNA-Seq by Expectation Maximization) value. Unpaired Student's t-test was used to analyze the differences between groups. **B.** Kaplan-Meier plots of OS of all patients divided by ITGB3 expression. **C-E.** Kaplan-Meier plots of OS of AML patients segregated by ITGB3 expression in favorable, intermediate and poor groups, respectively. **F-G.** Kaplan-Meier plots of OS of AML patients segregated by ITGB3 expression in FLT3 wild type and FLT3 mutation groups, respectively.

To investigate the prognostic significance of ITGB3 gene expression in human AML, we mined The Cancer Genome Atlas (TCGA) database which is available to public. The clinicopathological features of all AML patients are categorized in Table 1. We found that the patients with higher ITGB3 expression were older (median age, 57.8 vs 52.8 years; *p<*0.05) and had a higher proportion of patients with unfavorable cytogenetics (p=0.002) compared with patients who had lower ITGB3 expression. Moreover, we found patients with higher ITGB3 expression had lower blasts in peripheral and bone marrow, lower WBC counts, higher platelet counts compared with the patients with lower ITGB3 expression (*p<*0.05). When grouped with prognostic risk stratification of AML according to NCCN, the expression level of ITGB3 was significantly higher in poor group than in favorable and intermediate groups (Figure 1A).

**Table 1:** Relationship between ITGB3 expression and clinicopathological features of AML patients

Characteristic	No.	ITGB3 high	ITGB3 low	*p* value
No. of patients	173	87	86	
**Sex**				0.707
Male	93	48	45	
Female	80	39	41	
Median age, y (range)		57.8 (21-88)	52.8 (18-81)	0.043
**FAB classification, no.**				0.252
M0	16	12	4	
M1	42	19	23	
M2	39	18	21	
M3	16	5	11	
M4	35	18	17	
M5	18	10	8	
M6	2	1	1	
M7	3	3	0	
Not Classified	2	1	1	
**Cytogenetic abnormality, no.**				0.002
Favorable	32	7	25	
Intermediate	103	56	47	
Poor	36	23	13	
Unknown	2	1	1	
**FLT3 mutation rate (%)**		24/85 (27.9)	26/84 (31.3)	0.699
**Peripheral blast, % (range)**		61.2 (0-97)	70.4 (0-100)	0.006
**Bone marrow blast, % (range)**		74.3 (10-100)	84.9 (10-100)	0.010
**Median WBC count, ×10^9^ (range)**		26.3 (1-137)	49.5 (1-297)	0.001
**Median Hb concentration, g/dL**		9.8 (6-14)	9.4 (6-12)	0.295
**Median platelet count, ×10^9^ (range)**		80.5 (9-351)	47.5 (8-215)	0.000

Cytogenetic abnormality: according to NCCN guideline.

FAB classification, French-American-British classification; FLT3, Fms-like tyrosine kinase-3; WBC, white blood cell; Hb, hemoglobin.

### High level of ITGB3 expression is associated with shorter overall survival of AML patients, especially in patients with *FLT3-ITD* mutation

To determine the prognostic value of ITGB3 expression in AML patients, we evaluated the overall survival (OS) of all patients by Kaplan-Meier analysis. The median follow-up in this cohort was 557.4 days (0-2861 days). Cut-off value of ITGB3 expression (RNA-Seq by Expectation Maximization, RSEM) is set to −0.10 so that the numbers in two groups are similar [ITGB3 high group (n=81): −0.09 to 6.80; ITGB3 low group (n=82): −6.86 to −0.11]. As shown in Figure 1B, elevated ITGB3 expression was significantly correlated with shorter OS (*p<*0.05). Then we analyzed the OS depended on ITGB3 expression in each group according to prognostic risk stratification of NCCN. The OS was significantly worse when higher expression level of ITGB3 in poor prognostic group (Figure 1E), however, there was no correlation in favorable and intermediate groups (Figure 1C, 1D), Mutation of FLT3 was frequent and indicated poor prognosis in AML [1]. We thus compared the prognostic significance of ITGB3 on AML patients with or without FLT3 mutation. In FLT3 wild type AML patients, there was no difference of OS between two groups depends on the expression level of ITGB3 (Figure 1F), however, in patients who had normal cytogenetics with FLT3 mutation, the OS of patients with higher ITGB3 expression was significantly worse compared that of patients with lower ITGB3 (Figure 1G).

### Blocking integrin αvβ3 enhances leukemia sensitivity to sorafenib in BMSCs and OPN ligation

**Figure 2:**
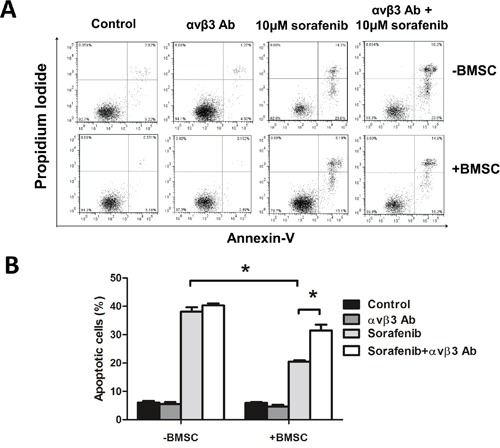
Blocking integrin αvβ3 increased leukemia sensitivity to sorafenib in BMSCs **A.** The apoptosis rate measured by flow cytometry by Annexin-V/PI double staining. MV4-11 cells were seeded onto the BMSC monolayer or cultured alone in 6-well plates with or without αvβ3 blocking antibody (1μg/ml) for 2 hours, sorafenib (10μM) was then added and cultured for 24 hours. Suspension cells were used for apoptosis assay. **B.** The percentage of apoptosis in each group. *p<0.05.

To figure out the function of ITGB3 in *FLT3-ITD* AML in bone marrow environment, we used BMSCs to mimic bone marrow environment. We co-cultured MV4-11 cells (a *FLT3-ITD* mutation AML cell line) with BMSCs and tested the sensitivity of sorafenib, a TKI proved by Food and Drug Administration for hepatocellular carcinoma and renal cell carcinoma. As showed in Figure 2A, MV4-11 was sensitive to sorafenib, by the 50% inhibitory concentration (IC-50) around 10μM (data not shown). Sorafenib induced apoptosis in MV4-11, the apoptotic rate was 38.15% (±1.48). When co-cultured with BMSCs, MV4-11 showed significantly reduced apoptosis rate under sorafenib treatment, the apoptosis rate was 20.54% (±0.44) (Figure 2A, 2B). This phenomenon could be explained by cell adhesion mediated drug resistance. However, when we blocked αvβ3 by pretreating with αvβ3 blocking antibody, this effect was significantly decreased. MV4-11 showed increased apoptosis rate after αvβ3 blocking (Figure 2A, 2B). We also detected lower Bcl-2 and higher Bax level when treated with sorafenib plus αvβ3 antibody in co-culture system (Supplementary Figure S1). These results indicating integrin αvβ3 trigger apoptosis resistance effect. Our previous work showed that bone marrow stromal cells secreted more OPN when co-cultured with MV4-11 cells (Zhaohua Shen, Oncology letters, 2016, in press). OPN was one of the ligand which could bind integrin αvβ3. We also tested the effect of OPN and αvβ3 in MV4-11. OPN could induce sorafenib insensitivity of MV4-11 cells in a dose-dependent manner (Supplementary Figure S2). These effects could be partly abrogated by αvβ3 blocking. These results suggested that integrin αvβ3 induced leukemia insensitivity to sorafenib when co-cultured with BMSCs as well as OPN ligation.

### OPN/αvβ3 activated β-catenin via PI3K/Akt/GSK3β signaling pathway

**Figure 3:**
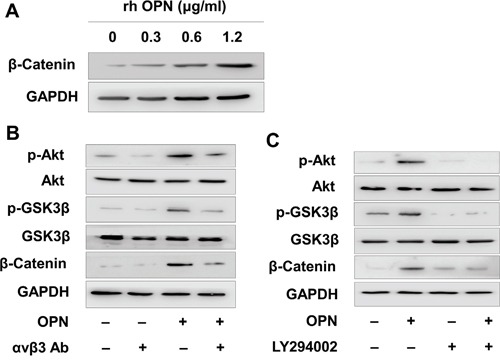
OPN/αvβ3 activated β-catenin via PI3K/Akt/GSK3β signaling pathway **A.** MV4-11 was treated by various amount of recombinant human OPN for 24 hours, then β-catenin was detected by Western blot assay. **B.** MV4-11 was pretreated by αvβ3 blocking antibody (1μg/ml) for 2 hours, then treated by OPN (1.2 μg/ml) for another 24 hours. The phosphorylation level of Akt and GSK3β and the expression of β-catenin were measured by Western blot analysis. **C.** MV4-11 was pretreated by PI3K inhibitor LY294002 (25μM) for 2 hours, then treated by OPN (1.2 μg/ml) for another 24 hours. The phosphorylation level of Akt and GSK3β and the expression of β-catenin were measured by Western blotting analysis.

It was reported that OPN/αvβ3 pathway promote a cancer stem cell-like phenotype in hepatocellular carcinoma cells. Because the Wingless-type (Wnt)/β-catenin is the most important cancer stem cell pathway, we hypothesized that αvβ3 could activated β-catenin in MV4-11 cells. We detected higher β-catenin level in OPN treated MV4-11 cells in a dose-dependent manner (Figure 3A). The elevated β-catenin was largely contributed by β-catenin in the nuclear (Supplementary Figure S3). Moreover, we observed higher phosphorylation of Akt, GSK3β and β-catenin in OPN treated MV4-11 (Figure 3B). This effect was largely abrogated when blocking αvβ3 before OPN ligation, indicating β-catenin elevation is αvβ3 dependent and through PI3K/Akt/GSK3β signaling pathway (Figure 3B). We also detected higher phosphorylation of Akt, GSK3β and β-catenin when co-culture MV4-11 with BMSCs, and αvβ3 antibody could partly reduce them (Supplementary Figure S4). To further validate PI3K/Akt/GSK3β contributes to β-catenin elevation and drug sensitivity, we used PI3K specific inhibitor LY294002. As we expected, p-Akt, p-GSK3β and β-catenin were decreased after PI3K inhibition (Figure 3C). Sorafenib sensitivity was increased after PI3K inhibition (Supplementary Figure S2). These results indicated that OPN/αvβ3 activated β-catenin and through PI3K/Akt/GSK3β signaling pathway.

### β-catenin maintained leukemia cell survival and contributed to sorafenib insensitivity

**Figure 4:**
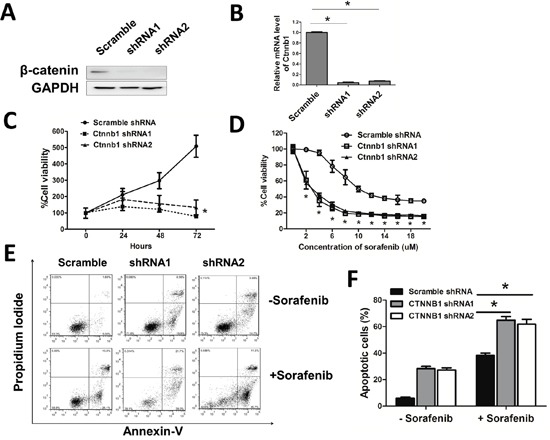
β-catenin maintained leukemia cell survival and contributed to sorafenib insensitivity **A**, **B.** β-catenin protein level and mRNA level were measured by Western blotting and real-time qPCR after *Ctnnb1* shRNA vectors infection and puromycin selection for 2 days. *p<0.05. **C.** Cell viability was measured by CCK-8 assay when cultured for 24, 48, 72 hours, respectively. *p<0.05. **D.** Cells were treated with different doses of sorafenib for 24 hours. Cell viability was then evaluated by CCK-8 assay. *p<0.05. **E.** Cells were treated with or without sorafenib (10μM) for 24 hours. **F.** The percentage of apoptosis in each group. *p<0.05.

To determine the effect of β-catenin in leukemia cells, we knocked down β-catenin in MV4-11 using lentiviral delivery of shRNA. To avoided off-target effect, we used two shRNAs which targeted different code sequence of *Ctnnb1* and a scramble shRNA as control. β-catenin was dramatically reduced in *Ctnnb1* knock down cells (sh-*Ctnnb1* cells) by western blotting and real time qPCR assay (Figure 4A, 4B). MV4-11 cell viability was decreased after *Ctnnb1* knocked down (Figure 4C). When exposed in different concentration of sorafenib, the cell viability was decreased gradually in each group. However, at the same concentration of sorafenib, the cell viability was higher in control cells compared with sh-*Ctnnb1* cells (Figure 4D). Sh-*Ctnnb1* cells underwent spontaneous apoptosis. When treated with 10μM sorafenib, the apoptosis rate of sh-*Ctnnb1* cells was higher compared with control cells, indicating β-catenin interference could enhance leukemia sensitivity in MV4-11 cells (Figure 4E, 4F).

## DISCUSSION

Integrins enhance cell-ECM interaction, intracellular signal transduction, contributing to tumor invasiveness and metastasis [14]. Integrin comprises of two subunits, alpha and beta chain, which form a heterodimer. Integrin beta3 (ITGB3) loss impairs AML survival and homing to endosteum [13]. ITGB3 has prognostic significance in AML especially for unfavorable group and patients with *FLT3-ITD* mutation according to our data. Higher level of ITGB3 implies older and higher frequency of poor cytogenetics. However, there are significant lower WBC counts, lower blast and higher platelet accompanied with higher level of ITGB3, indicating ITGB3 signaling is not associated with higher leukemia proliferation rate. In myeloid cells, ITGB3 heterodimerizes with ITAV and interact with ECM [15]. We also analyze ITGAV and its clinical relevant, however, there is no significant relevant in TCGA database (data not shown). Whether ITGAV has clinical relevant needs further investigation.

Integrin αvβ3 could bind arginine-glycine-aspartate (RGD)-containing components of matrix, such as OPN, vitronectin and fibronectin, trigger intracellular signal [16]. OPN/αvβ3 was found to drive tumor stemness and resistant to TKI inhibition in solid tumor [17, 18]. In this study, we give evidence that integrin αvβ3 could alternatively enhance β-catenin activation in AML. β-catenin signaling contributes to leukemia stem cell phenotype and influences drug sensitivity in leukemia [19–21]. It is also an independent prognostic indicator in AML [22]. β-catenin could be activated in several ways. First, several Wnt ligands are constitutive expressed in most AML cells and bone marrow stromal cells, leading to Wnt/β-catenin signaling in AML [19, 23]. Second, *FLT3-ITD* positive AML has ligand independent activated form of FLT3 receptor tyrosine kinase and causes increased activated form of β-catenin [24]. Here, we give evidence that integrin αvβ3 could alternatively enhance β-catenin activation through PI3K/Akt/GSK3β cascade on linking OPN from microenvironment, giving an another interpretation on β-catenin activation in *FLT3-ITD* mutated AML.

*FLT3-ITD* positive AML patients might benefit from TKIs combining with integrin αvβ3 blocker and β-catenin inhibitors. Several αvβ3 antibody and β-catenin inhibitors are under clinical trial in several cancer types [25–30]. Based on our study, these compounds might be considered as new pharmaceuticals for therapy of AML with *FLT3-ITD* mutation combined with TKIs.

**Figure 5:**
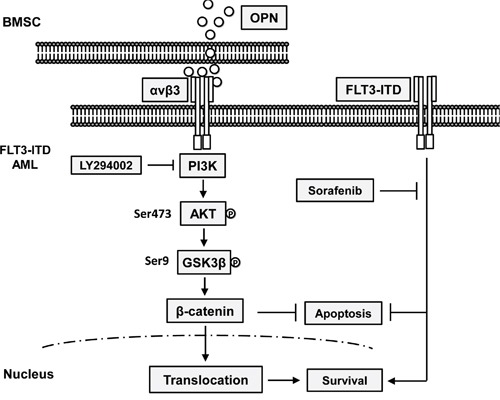
A schematic drawing of Integrin αvβ3/PI3K/Akt/GSK3β/β-catenin axis for microenvironment mediated TKI insensitivity in FLT3-ITD AML cells

Collectively, we demonstrated that integrin β3 has prognostic significance in AML especially in cytogenetic-normal AML with *FLT3-ITD* mutation. Integrin αvβ3/PI3K/Akt/GSK3β/β-catenin axis is crucial for microenvironment mediated TKI insensitivity in *FLT3-ITD* AML cells (A schematic drawing is shown in Figure 5). Our data provides a new mechanistic basis for integrin αvβ3 signaling in drug insensitivity in bone marrow microenvironment. Targeting integrin αvβ3/PI3K/Akt/β-catenin signaling might be helpful improving the prognosis of *FLT3-ITD* mutated AML patients.

## MATERIALS AND METHODS

### Clinical data

The Cancer Genome Atlas (TCGA) dataset for AML are available on https://tcga-data.nci.nih.gov/tcga/. High-throughput sequencing data of transcriptome and clinical data were retrieved and analyzed.

### Cell line and cell culture

The MV4-11 human acute myeloid leukemia cell line (AML-M5, *FLT3-ITD* mutation) was obtained from the American Type Culture Collection. Cells were cultured in suspension in IMDM media (Hyclone, USA), supplemented with 10% fetal bovine serum (Gibco, USA) plus 1% penicillin/streptomycin (Hyclone, USA) at 37°C in a humidified 5% CO_2_ incubator. Cells were passaged every 3 days.

### Isolation and culture of bone marrow stromal cells

Bone marrow aspirates were obtained from healthy volunteers in Department of Hematology, Xinqiao Hospital after written informed consent was obtained. This study was approved by the Ethical Committee of Xinqiao Hospital and carried out in accordance with the Declaration of Helsinki. BMSCs were isolated and cultured as previously described [31]. Briefly, Mononuclear cells from healthy donors after informed consent were isolated with a density gradient centrifugation (Ficoll-Paque, GE Healthcare, NJ) and cultured in Dulbecco's minimal essential medium (DMEM) with low glucose, supplemented with 10% fetal bovine serum plus antibiotics as mentioned above, and maintained at 37°C in a humidified 5% CO_2_ incubator. The nonadherent cells were removed 24 hours later. When 80–90% confluency, the adherent cells were trypsinized and passaged. BMSCs were used in the following experiments at passage 3 or 4.

### Drugs, chemicals and antibodies

Sorafenib was obtained from Bayer Pharmaceutical Corporation and was dissolved in dimethylsulfoxide (DMSO) (Sigma, St.Louis, Mo), stored in −20°C. For apoptosis assay, the final concentration of sorafenib was 10μM, and DMSO concentration was 0.01%. The αvβ3 blocking antibody was purchased from NOVUS Biologicals (Littleton, CO). Recombinant OPN was purchased from PeproTech (Rocky Hill, NJ). PI3K inhibitor LY294002 was purchased from Selleck Chemicals (Houston, TX). Primary rabbit anti-human antibodies against β-catenin, phospho-Akt (Ser473), Akt, phospho-GSK3β (Ser9), GSK3β were purchased from Cell Signaling Technology, Inc (Beverley, MA). Primary rabbit anti-human antibodies against Bcl-2 and Bax were purchased from Abcam (Cambridge, MA). Rabbit anti-human antibody against GAPDH and Histone H3, horseradish peroxidase-linked anti-rabbit secondary antibody were purchased from Bioworld Technology (Minneapolis, MN).

### Assessment of apoptosis by flow cytometry

Apoptosis was measured based on flow cytometry by annexin V-fluorescein isothiocyanate (FITC) and propidium iodide (PI) double staining using the FITC Annexin V Apoptosis Detection Kit (BestBio, Shanghai, China), according to the manufacturer's instructions. Flow cytometry was carried out using MoFlo XDP system (Beckman Coulter, Pasadena, CA). Data were analyzed by FlowJo software (Treestar Inc). In co-culture experiments, MV4-11 cells were seed onto the BMSCs monolayer in 6-well plates, then cells were treated with or without αvβ3 blocking antibody (1μg/ml) for 2 hours, sorafenib was then added and cultured for 24 hours. After that, suspension cells were removed by gently shaking and used for apoptosis assay.

### Lentiviral shRNA production and infection

Scramble shRNA (Sequence: TTCTCCGAACGTGTCACGTCTCGAGACGTGACACGTTCGGAGAA) or two *Ctnnb1* shRNAs (Sequence 1: TTGGAATGAGACTGCTGATCTCGAGATCAGCAGTCTCATTCCAAGC; sequence 2: GTTATCAGAGGACTAAATACTCGAGTATTTAGTCCTCTGATAAC) were cloned to GV112 vector (Genechem, Shanghai) carrying puromycin resistance gene. GV112-shRNA vector, VSV-G (Addgene plasmid 8454, MA), and psPAX2 (Addgene plasmid 12260, MA) were co-transfected at a 3:1:2 ratio into 293T packaging cells using polyethylenimine (PEI) linear (Polysciences Inc). The medium was replaced 8 hours post-transfection and lentiviral supernatants were harvested at 48 hours. MV4-11 cells were plated at 2 × 10^5^ cells/well on 6-well plates overnight and added with lentiviral supernatants in the presence of 5 μg/mL polybrene (Sigma-Aldrich) with spin infection at 2,500 rpm and 32°C for 90 minutes. Cells were incubated for another 4 hours at 37°C incubator. After changing media, infected MV4-11 cells were cultured 48 hours and selected in 2 μg/mL puromycin for at least 2 days. Then cells were ready for the following experiments.

### Determination of cell viability

Infected MV4-11 were seeded in 96-well plates at a density of 5×10^3^ cells per well, cells were treated with various concentration of sorafenib for 24 hours. Cell viability was assessed using the Cell-Counting Kit-8 (CCK-8) (Dojindo, Kumamoto, Japan) according to the manufacturer's instructions. Briefly, after treatment, 10 μl CCK-8 was added to each well and incubated at 37°C for 1 hour. The optical density was read at a wavelength of 450 nm with a microplate reader (BioTek, USA). Cell viability was calculated as the following formula: optical density of treated group/control group×100%.

### Immunoblotting

MV4-11 cells were collected and total protein was lysed in cold radioimmunoprecipitation assay (RIPA) buffer (50 mM Tris [pH8], 150mM NaCl, 1% NP-40, 0.5% sodium deoxycholate, 0.1% SDS), containing protease and phosphatase inhibitors (Bayotime, Shanghai, China). In certain experiment, nuclear and cytoplasmic protein was extracted using Nuclear and Cytoplasmic Protein Extraction Kit (Bayotime, Shanghai, China). Protein samples were resolved by 10% SDS-polyacrylamide gels and transferred to polyvinylidene fluoride membranes (PVDF, Millipore Corporation, MA). PVDF membranes were blocked in Tris-buffered saline (TBS; 25 mM Tris, 0.15 M NaCl; pH 7.2) containing 5% nonfat dry milk and 0.1% Tween-20 for 1 hour and then incubated overnight in 4°C with various primary antibodies. After washing with TBST (TBS with 0.1% Tween-20), blots were incubated with horseradish peroxidase-linked secondary antibody at room temperature for 1 hour. Blots were developed using WesternBright ECL western blotting detection kit (Advansta Inc., Menlo Park, CA).

### Real-time quantitative PCR (qRT-PCR)

Total RNA was extracted from cultured cells using TRIzol reagent (Invitrogen, Carlsbad, CA) according to the manufacturer's instructions. QRT-PCR was performed using SYBR Premix ExTaq (Takara, Japan) and an Applied Biosystems 7500 Fast real-time PCR system according to the manufacturer's instructions. The sequences of primers used in this study were listed in Table 2. The PCR cycling conditions were as follows: 95°C for 10 min, followed by 40 cycles of 95°C for 15s and 60°C for 35s. Beta-actin (*Actb*) was used as an internal normalization control. Data analyses were performed using the comparative CT (ΔΔCT) method for calculating relative gene expression.

**Table 2:** The primer sequences used in the real time PCR experiment

Gene names	Forward	Reverse
Ctnnb1	5′-AAAGCGGCTGTTAGTCACTGG-3′	5′-CGAGTCATTGCATACTGTCCAT-3′
beta-actin	5′-CGTGCGTGACATTAAGGAGAAGC-3′	5′-CGGACTCGTCATACTCCTGCTTG-3′

### Statistical analysis

Statistical analysis was conducted by SPSS software, version 18.0 (SPSS Inc., Chicago, IL, USA). The normality distribution test was performed with the Kolmogorov-Smirnov test. Clinical features of sex and FLT3 mutation rate were compared between ITGB3 high and low groups by the Pearson χ2 test. Clinical features of FAB classification and Cytogenetic abnormality were compared between two groups using Fisher's exact test. Other clinical features of age, peripheral blast, bone marrow blast, WBC count, Hb concentration and Platelet count between two groups were compared using the unpaired two-tailed Student's t-test. Overall survival curves were plotted according to the Kaplan-Meier methods with the log-rank test applied for comparison. Unpaired two-tailed Student's t-test for two groups was applied in the *in-vitro* experiments. All values are presented as mean±SEM. A P value<0.05 was considered statistically significant (assigned as *).

## SUPPLEMENTARY FIGURES


