# Supplementary material for: Integrin alphavbeta3 enhances β-catenin signaling in acute myeloid leukemia harboring Fms-like tyrosine kinase-3 internal tandem duplication mutations: implications for microenvironment influence on sorafenib sensitivity
Source: Oncotarget. 2016 May 26;7(26):40387–97. doi: 10.18632/oncotarget.9617 (PMC5130015; doi:10.18632/oncotarget.9617)
Supplement: Supplementary file 1 [file oncotarget-07-40387-s001.pdf]

Integrin alphavbeta3 enhances β-catenin signaling in acute myeloid leukemia harboring Fms-like tyrosine kinase-3 internal tandem duplication mutations: implications for microenvironment influence on sorafenib sensitivity

SUPPLEMENTARY FIGURES

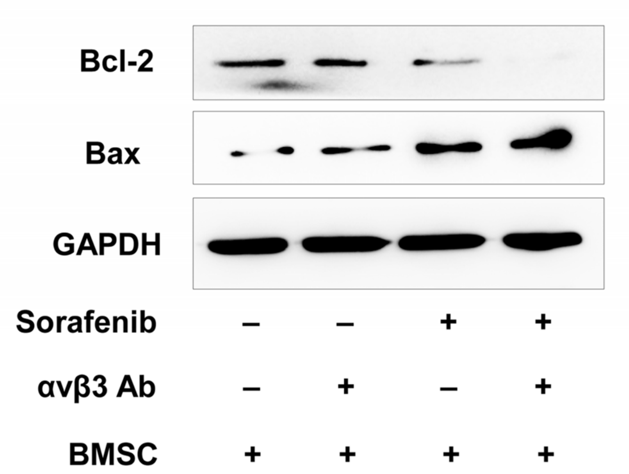

Supplementary Figure S1: Detection of Bcl-2 and Bax by western blot in MV4-11 cells co-cultured with BMSCs. MV4-11 cells were seeded onto the BMSC monolayer in 6-well plates with or without αvβ3 blocking antibody (1μg/ml) for 2 hours, sorafenib (10μM) was then added and cultured for 24 hours. Suspension cells were collected and used for western blot assay.

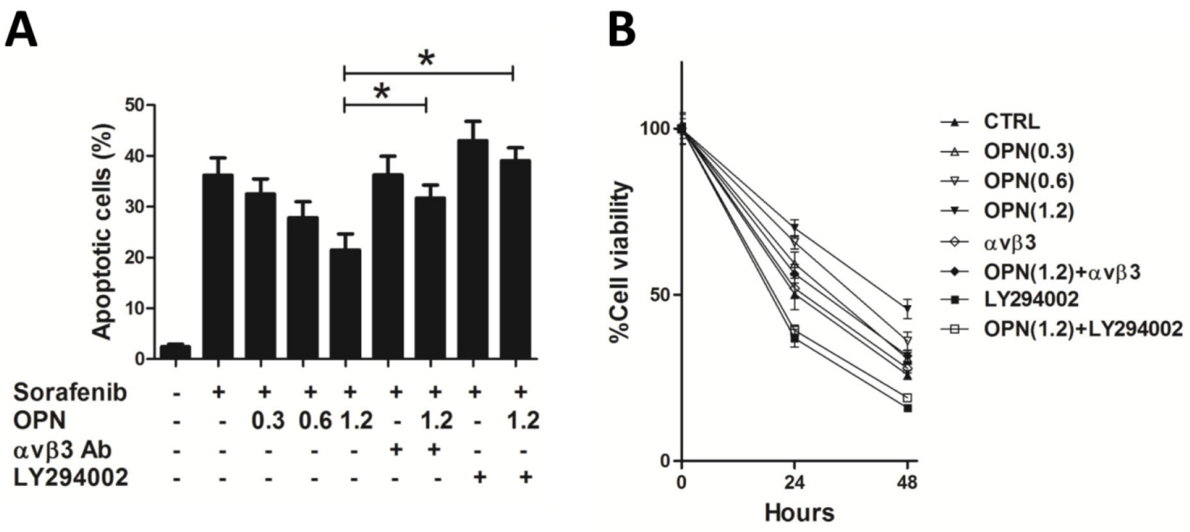

Supplementary Figure S2: Integrin αvβ3/ PI3K contribute to sorafenib insensitivity of MV4-11 cells on OPN ligation. MV4-11 was treated by indicate amount of recombinant human OPN (μg/ml) with or without αvβ3 blocking antibody (1μg/ml)/ PI3K inhibitor LY294002 (25μM) for 24 hours, sorafenib (10μM) was then added and cultured for another 24 hours to 48 hours. Cells were collected and used for apoptosis assay at 24 hours **A.** and cell viability assay at 24, 48 hours **B.**

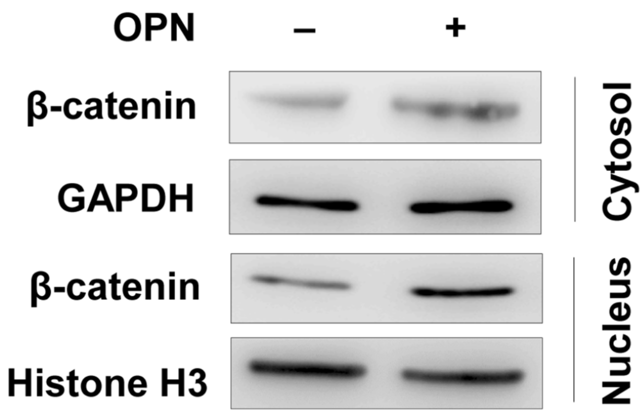

Supplementary Figure S3: Increased β-catenin nuclear translocation on OPN ligation. MV4-11 cells were treated with OPN (1.2 μg/ml) for 24 hours, then cells were collected. Nuclear and cytoplasmic protein was extracted and used for western blot assay.

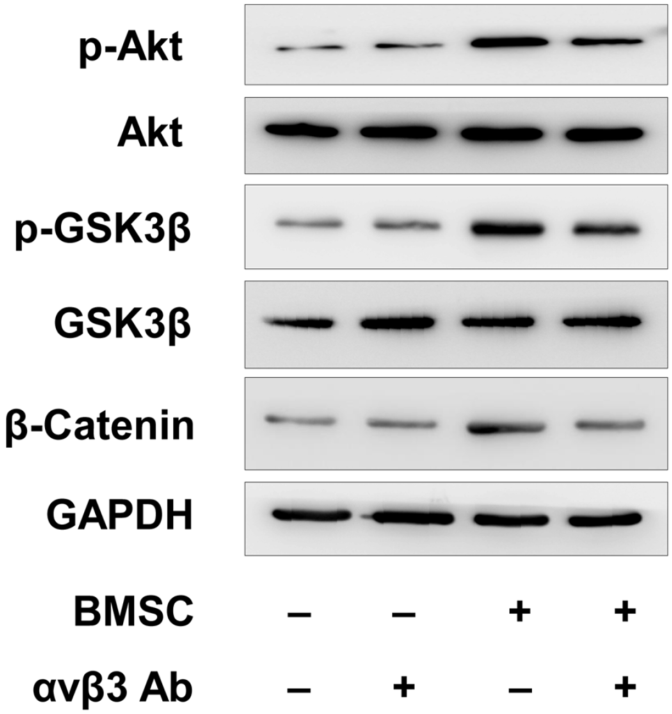

Supplementary Figure S4: Integrin αvβ3 contributes to Akt/ GSK3β/ β-catenin activation in MV4-11 cell line when co-cultured with BMSCs. MV4-11 cells were seeded onto BMSCs with or without αvβ3 blocking antibody (1μg/ml) for 24 hours, suspension cells were then collected and used for western blot assay.
